# Digital health for optimal supportive care in oncology: benefits, limits, and future perspectives

**DOI:** 10.1007/s00520-020-05539-1

**Published:** 2020-06-12

**Authors:** M. Aapro, P. Bossi, A. Dasari, L. Fallowfield, P. Gascón, M. Geller, K. Jordan, J. Kim, K. Martin, S. Porzig

**Affiliations:** 1 Medical Oncology, Genolier Cancer Center, Clinique de Genolier, Genolier, Switzerland; 2 Institut Multidisciplinaire d’Oncologie (IMO), Clinique de Genolier, Case Postale (PO Box) 100, 1 Route de Muids, CH-1272 Genolier, Switzerland; 3 Department of Medical Oncology, University of Brescia, Brescia, Italy; 4 Department of Gastrointestinal Medical Oncology, Division of Cancer Medicine, MD Anderson Cancer Center, Houston, TX USA; 5 Sussex Health Outcomes Research & Education in Cancer (SHORE-C), Brighton & Sussex Medical School, University of Sussex, Brighton, UK; 6 Department of Hematology-Oncology, Hospital Clínic de Barcelona, University of Barcelona, Barcelona, Spain; 7 Gynecologic Oncology, Department of Obstetrics, Gynecology and Women’s Health (OBGYN), University of Minnesota, Minneapolis, MN USA; 8 Department of Medicine, Haematology, Oncology and Rheumatology, Heidelberg University Hospital, Heidelberg, Germany; 9 Medical Oncology, Yale University School of Medicine, New Haven, CT USA; 10 Gyneco-oncology, Barnes-Jewish Hospital, St. Louis, MO USA; 11 Medical Oncology, Fox Chase Cancer Center, Philadelphia, PA USA

**Keywords:** Digital therapeutics, Integrative oncology, Symptom monitoring, Self-management, Patient-reported outcomes, eHealth

## Abstract

**Background:**

Digital health provides solutions that capture patient-reported outcomes (PROs) and allows symptom monitoring and patient management. Digital therapeutics is the provision to patients of evidence-based therapeutic interventions through software applications aimed at prevention, monitoring, management, and treatment of symptoms and diseases or for treatment optimization. The digital health solutions collecting PROs address many unmet needs, including access to care and reassurance, increase in adherence and treatment efficacy, and decrease in hospitalizations. With current developments in oncology including increased availability of oral drugs and reduced availability of healthcare professionals, these solutions offer an innovative approach to optimize healthcare resource utilization.

**Design:**

This scoping review clarifies the role and impact of the digital health solutions in oncology supportive care, with a view of the current segmentation according to their technical features (connection to sensors, PRO collection, remote monitoring, self-management in real time…), and identifies evidence from clinical studies published about their benefits and limitations and drivers and barriers to adoption. A qualitative summary is presented.

**Results:**

Sixty-six studies were identified and included in the qualitative synthesis. Studies supported the use of 38 digital health solutions collecting ePROs and allowing remote monitoring, with benefits to patients regarding symptom reporting and management, reduction in symptom distress, decrease in unplanned hospitalizations and related costs and improved quality of life and survival. Among those 38 solutions 21 provided patient self-management with impactful symptom support, improvement of QoL, usefulness and reassurance. Principal challenges are in developing and implementing digital solutions to suit most patients, while ensuring patient compliance and adaptability for use in different healthcare systems and living environments.

**Conclusions:**

There is growing evidence that digital health collecting ePROs provide benefits to patients related to clinical and health economic endpoints. These digital solutions can be integrated into routine supportive care in oncology practice to provide improved patient-centered care.

## Introduction

The International Agency for Research on Cancer estimated that in 2018, there were 18.1 million new cancer cases worldwide and 9.6 million cancer-related deaths [1]. A global surveillance report suggests a trend toward increased survival [2], with some cancers progressing to chronicity. However, the total burden of new cancer cases is increasing, and new therapies are generally more costly [3]. Additionally, more drugs are available in oral formulations for home administration, with reduced face-to-face surveillance by healthcare professionals (HCPs). Novel approaches for optimal patient management that allow containment of healthcare costs are urgently needed [4].

The new approaches should focus on patient-centered care with integration of tumor-directed treatment and patient-directed supportive and palliative care throughout the disease journey [5, 6]. The goals of management are to achieve improvements in not only overall survival (OS) but also patient-reported outcomes (PROs) such as quality of life (QOL) [7], fewer emergency department visits, and self-reported improvements in symptoms [7, 8].

The intensive development over recent years of therapies with novel mechanisms of action, including molecular-targeted therapies, immuno-oncology therapies, and precision radiation oncology, has transformed the oncology treatment landscape [9, 10]. These advances have increased the complexity of treatment (combination of therapies) and required modifications in the patient pathway (oral treatment intake at home versus hospitalization) to ensure quality care. The real-world toxicity profile of novel agents may not always correlate with that observed in clinical trials and may result in unanticipated toxicities [11, 12]. Increased availability of oral therapies for home administration results in less healthcare supervision during treatment, whereas the prolonged use of such treatments as long-term maintenance may be associated with the emergence of new toxicities [13]. Therefore, careful monitoring of adverse events (AEs) during self-administration of treatments at home is becoming essential to facilitate prompt intervention to reduce their severity and duration.

Patients must therefore manage symptoms and treatment-related side effects without direct medical supervision; home administration of anticancer treatments also increases the chance of nonadherence and administration errors by patients [14]. With immunotherapeutic treatments, the timely identification of toxicities is crucial since many symptoms may improve with prompt intervention [15]. Additionally, a potential shortage in oncology services and workforce linked to the increasing cancer incidence and complexity of cancer treatments [16] has highlighted the need for new strategies to ensure that all patients receive optimal treatment and care throughout the continuum of disease.

Advances in digital communications and medical technologies have led to the digitalization of healthcare [17]. Increased access and uptake of such technologies among physicians and patients yields large amounts of potentially usable data, which, in the context of electronic health records (EHRs), forms an important part of physicians’ decision-making. Self-reported data is extensively used in healthcare. Patient-level data provide real-world medical information, with opportunities for improved clinical decision-making, patient empowerment, improved health outcomes, and cost reductions [18–20]. However, patient confidentiality and compliance with local and global data privacy regulations need to be ensured.

### Digital health definitions with focus on digital therapeutics

**Fig. 1:**
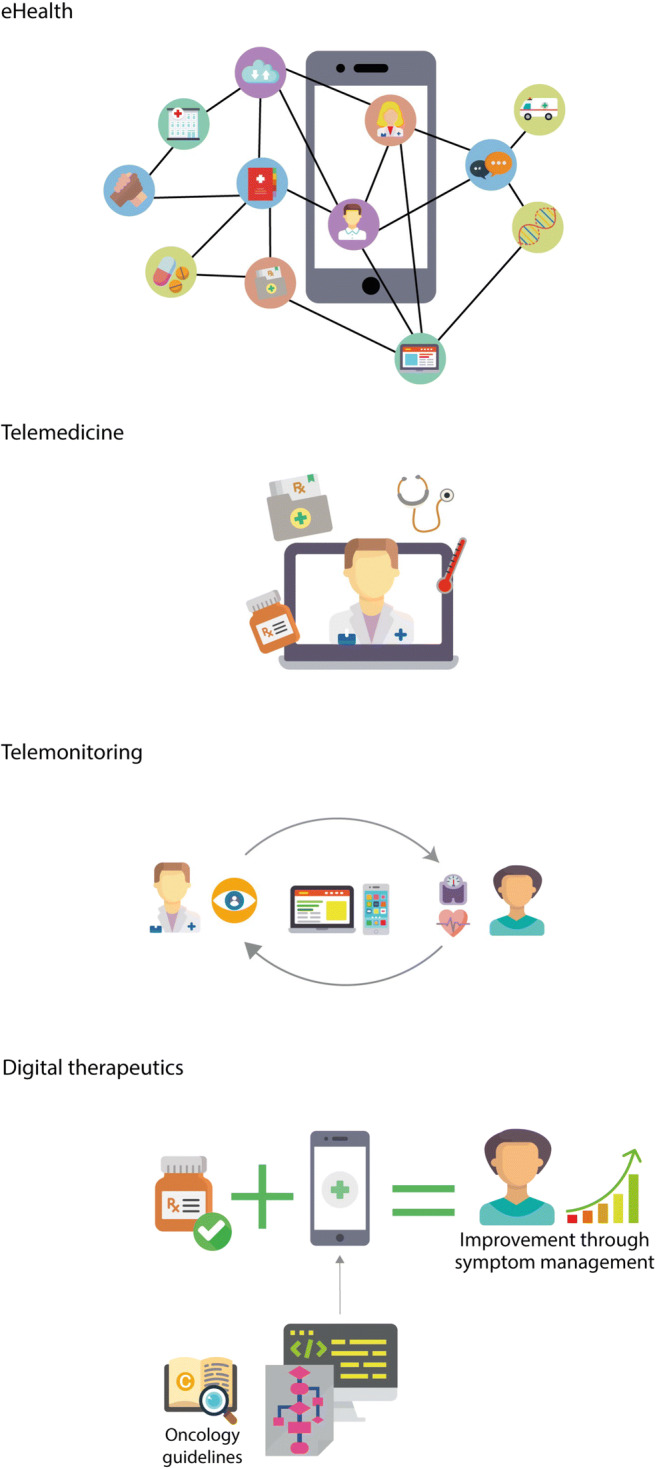
Digital health definitions

Digitalized healthcare comprises eHealth, telemedicine, telemonitoring, and digital therapeutics (Fig. 1).

The terms digital health, telehealth, and eHealth are interchangeable and are defined as the provision of healthcare services supported by telecommunications or digital technology to improve or support healthcare services. eHealth solutions can be part of each step of the healthcare process (i.e., prevention, diagnosis, decision-making, treatment/intervention, and follow-up).

Telemedicine represents medical services provided remotely to patients by HCPs using telecommunications platforms. Healthcare activities, such as patient evaluation, diagnosis, or treatment, are performed by HCPs without the need for inpatient consultation, although the legal status of such consultations varies according to jurisdiction [21].

Telemonitoring is the use of digital technology to frequently or continuously monitor patients’ vital signs or any other symptoms. The information is assessed remotely by HCPs to inform the patient and caregivers about the actions needed for appropriate symptom management and treatment advice.

Digital therapeutics embed algorithms based on medical guidelines and best practices, which transform collected data into actionable insights, with the objective to bring value to evidence-based clinical outcomes (from clinical studies or real-world evidence). They may be used alone or in conjunction with drugs and medicinal products, medical devices, or other therapies, to enhance and support medical treatment. According to the risk level of the embedded algorithms, the digital therapeutics may be classified as medical devices. Depending on the regulatory status, they may be used on prescription only (prescription digital therapeutics).

A further technology of relevance to the broad concept of digitalized healthcare is artificial intelligence with capabilities of machine learning, which may be defined as the use of computer algorithms to make successful predictions about future events based on past experiences [22].

From a health outcomes perspective, digital health can be grouped into solutions connected to sensors or not and that capture ePROs to allow patient monitoring only or those that allow patient monitoring and symptom management by HCPs, covering remote areas, or symptom management by the patients themselves with or without real-time decision support for self-management. Patients receive individualized guidance, from a simple recommendation to call their HCP, to a suggestion to begin a specific treatment intake.

### Supportive care for cancer patients definition and unmet needs

The Multinational Association of Supportive Care in Cancer defines supportive care in cancer as “the **prevention and management of the adverse effects of cancer and its treatment**. This includes management of physical and psychological symptoms and side effects across the continuum of the cancer experience from diagnosis through treatment to post-treatment care. Enhancing rehabilitation, secondary cancer prevention, survivorship, and end-of-life care are integral to supportive care.”(About MASCC. mascc.org/about-mascc. Accessed January 11, 2019). Whereas there has been significant progress in anticancer treatment, improvements for optimal supportive care are still needed at all stages of the cancer treatment pathway [5]. Currently, supportive care interventions’ assessment of patient QOL and medical outcomes remains limited, and QOL endpoints are insufficiently reported for clinical trials of novel therapies [23].

A number of evidence-based supportive care guidelines have been developed, but their implementation in routine clinical practice is suboptimal and the opportunity to improve control of symptoms is often forfeited [24]. This highlights the need for more optimal use of guidelines, for personalized and patient-centered care that is delivered in a timely manner.

Digital solutions present an opportunity to address certain unmet needs in prevention or management of adverse events in patients with cancer including (1) increased communication between patients, providers, and their communities [18]; (2) education of patients and caregivers; (3) integration of standard clinical assessments with PROs measured during routine clinical practice; (4) help of patients in monitoring their respective conditions [18]; (5) improved patient empowerment and self-management; and (6) improved evidence from clinical trials on the basis of PRO endpoints in studies evaluating anticancer treatments and prospective evaluations of supportive care interventions and real-world efficiency of care for cancer patients.

The objectives of the present review are to evaluate the state of digital health solutions in oncology supportive care allowing collection of ePRO and focused on symptom management and to identify benefits and limitations.

## Methods

**Fig. 2:**
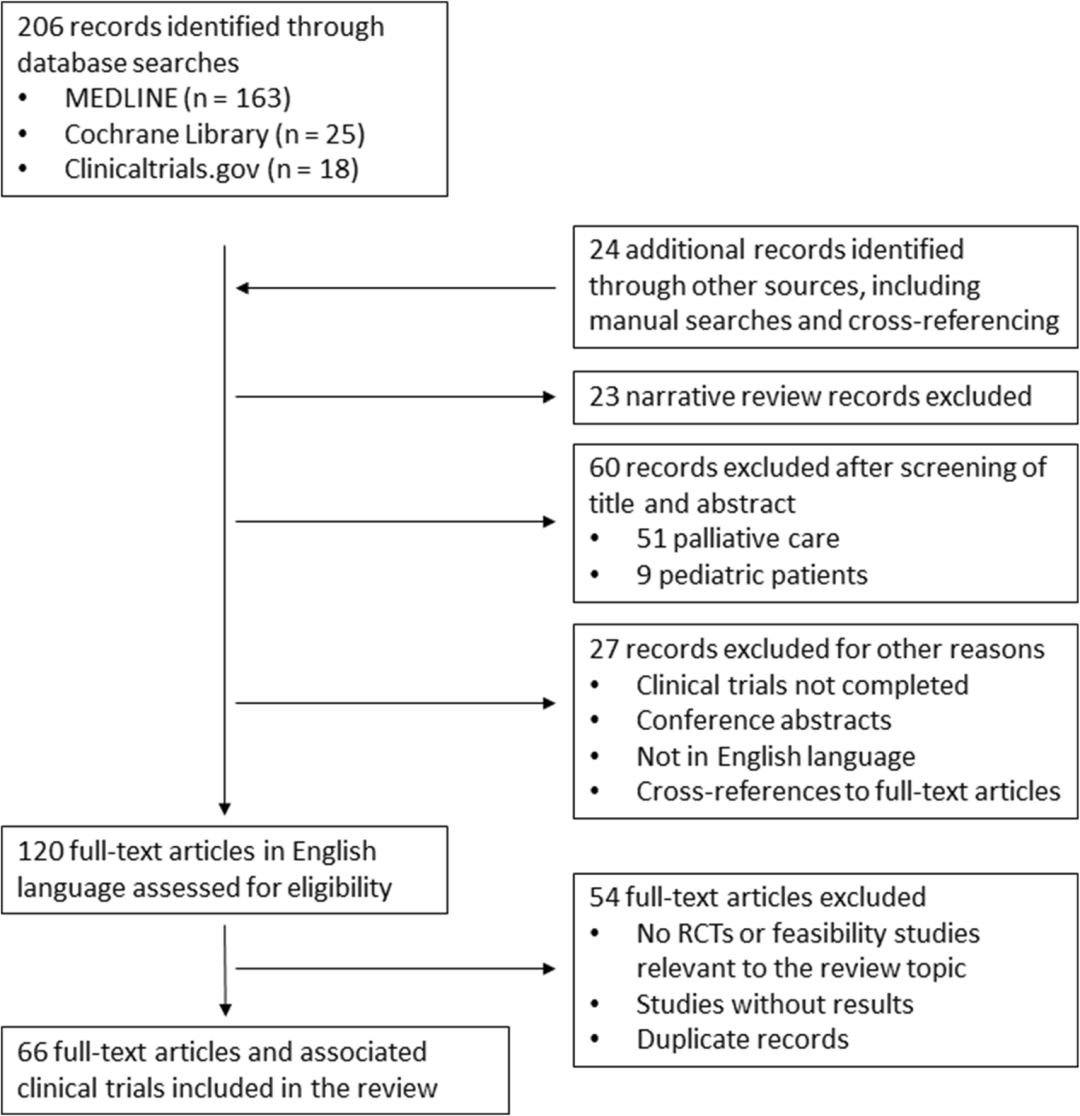
PRISMA statement. PRISMA Preferred Reporting Items for Systematic Reviews and Meta-Analyses, RCT randomized controlled trial

Guidance of the Preferred Reporting Items for Systematic Reviews and Meta-Analyses (PRISMA) statement was followed in the conduct of this study (Fig. 2).

### Search strategy

The MEDLINE Public Library of Medicine (PubMed) database and the Cochrane Library were explored from December 1, 2008, to November 30, 2018, for relevant studies using the following search terms: (1) MEDLINE, “cancer or oncology” AND “telehealth or eHealth” AND “symptom management” or “symptom monitoring”; (2) Cochrane Library (title abstract keywords), “cancer or oncology” AND “telehealth or eHealth” AND “symptom”. Clinicaltrials.gov search was performed using the following search strategy: “cancer or oncology” (condition or disease) AND “telehealth or eHealth” (other terms) AND “symptom” (outcomes measures).

### Study eligibility criteria (inclusion/exclusion)

Screening of publication was done by 2 researchers on titles and abstracts and then full-text to ensure eligibility to the following criteria.

#### Inclusion criteria

Adult cancer patients, all randomized controlled trials (RCTs) or feasibility and pilot studies that evaluated the effectiveness of telehealth or eHealth solutions in supportive cancer care were eligible for inclusion in English language.

#### Exclusion criteria

Studies involving pediatric patients and those evaluating solutions at the palliative phase were excluded.

For results retrieved from clinicaltrials.gov, not completed studies or studies without published results were excluded.

Retrieved studies were reviewed, and those evaluating solutions at palliative latest phase of cancer were removed from the analysis.

### Outcomes of interest selected and assessed

Outcomes of interest were as follows for each digital solution identified: description of the digital solution including PRO for supportive care in oncology, with remote monitoring, with/without patient automated symptoms self- management, its benefits, limitations, drivers of and barriers to adoption; unmet needs; PRO data including QOL outcomes; AE incidence, severity, and management; emergency room (ER) admissions and hospitalizations; health resource utilizations; and survival outcomes including OS.

### Data collection and analysis

Search results were critically analyzed by the authors for relevance to the focus of this review. Two researchers extracted the data. The authors analyzed systematically according to outcomes of interests detailed above the study results to critically discuss the impact on outcomes of the various digital solutions.

## Results

A total of 206 articles have been identified through databases searches in Medline, Cochrane, and Clinicaltrials.gov. Twenty-four (24) additional records were provided from other sources (manual search, cross-references). We excluded narrative reviews (23), publications which titles and abstracts were about pediatric population or focused on palliative care phase of cancer (60), and other records (27) (not completed results in clinical trials, conference abstracts, not in English language, cross references to full-text articles).

Regarding the 120 selected articles, another 54 full-text articles were excluded because of absence of study results, duplicates, or design (exclusion when not a RCT nor a feasibility study).

Finally, 66 full-text articles and associated clinical trials are included in this review.

### Digital health solutions in oncology

The review results outlining the status of clinical evidence regarding digital health solutions that collect ePRO for supportive care in oncology are summarized in Table 1 [7, 8, 25–86]. These 38 digital solutions can be classified into 2 main categories: the first, 17 digital solutions based on PRO collection only, and the second, 21 digital solutions providing also self-management. The key findings are summarized according to outcome.

**Table 1:** Description of digital solutions for supportive care in oncology with remote monitoring with/without patient automated symptoms self- management

Digital solutions description			Study type^a^	*N*	Tumor type/inclusion criteria	Results		Reference
Name	Remote symptom monitoring (mobile, web, phone based)	Symptom management with patient automated self-management				Patients	HCPs	
ASyMS©	Yes Mobile phone-based Rate the severity and bother of each symptom (CTCAE based) though 10 specific chemotherapy-related symptoms questionnaires (ie, nausea, vomiting, diarrhea, constipation, hand-foot syndrome, mucositis, paresthesia, flu-like symptoms, fatigue, and pain).	Yes^b^ Mobile phone-based Patients will immediately receive automated, evidence-based self-care advice on the basis of their symptom reports	RCT	112	Breast, lung, colorectal receiving CT	- Significantly less fatigue in the intervention group and less hand-foot syndrome in the control group - Improved communication with HCPs; improved symptom management; reassuring	- Useful for symptom management	Kearney [25], Maguire [26], McCann [27]
			Feasib., pilot	16	Lung receiving RT	- Less anxiety and drowsiness; improved self-care efficacy - Real-time symptom reporting; reassuring; fast HCP response to alerts	-*Positive*: generation of real-time alerts; self-care advice *-Negative*: questionable clinical use of alerts; increased workload	Maguire [28]
			Feasib., pilot	17	Hematologic receiving CT	- Feasible. Easy to use, reassuring; increased health awareness and empowerment; improved execution of self-care activities; improved communication with HCPs and family/friends; improved AE management - System limitations: inadequate grading scale for AEs; unclear language; limited AEs; less beneficial for patients with few AEs; inaccurate AE reporting by patients to avoid generating alerts	–	Breen [29]
			RCT in progress	222	Hematologic receiving CT	–	–	Breen [30]
			Feasibility to assess the ASyMS technological readiness before the RCT (Maguire [32])	64/	Breast, colorectal, hematologic receiving first-line CT	-Feasible. High compliance in all countries and all cancer types	- Technical issues with the Web-based platform. Resolved with additional training of physicians - Modifications in ASyMS: longer time frame to provide feedback; changes in symptom algorithm - ASyMS not feasible in 2 centers due to organizational issues: lack of staff and technology connectivity	Furlong [31]
			RCT in progress	1108	Breast, colorectal, hematologic receiving first-line CT	–	–	Maguire [32]
Automated voice response (AVR) system	Yes Phone-based Symptom management toolkit, completed a baseline interview. Symptoms questionnaires about: fatigue, pain, insomnia, poor appetite, constipation, nausea/vomiting, anxiety, cough, depression, diarrhea, mouth sores, shortness of breath, peripheral neuropathy, difficulty remembering, and weakness.	Yes (Paper-based information for symptom management; phone-based calls for adherence management) Weekly AVR calls	Pilot (AVR monitoring vs AVR + symptom and adherence management vs AVR + adherence management)	119	Solid tumor	- Symptom severity decreased similarly in all groups. No difference in adherence to oral chemotherapy treatment between groups	–	Spoelstra [33]
AWARE	Yes Phone-, wearable sensor-, and Fitbit-based passive data collection and PROs: pain, fatigue, feeling disconnected from others, trouble concentrating or remembering things, feeling sad or down, feeling anxious or worried, not enjoying things, feeling irritable, shortness of breath, numbness or tingling, nausea, and poor appetite.	No	Feasib. (Passively collected data vs PROs)	14	Gastrointestinal receiving CT	- Feasible; passively collected data during CT correlated with PRO scores with high accuracy	–	Low [34]
Bioconnect	Yes Web-based app Weekly self-scoring of 13 common patient symptoms among which: fever, shivers, a brutal asthenia, a decrease in urine volume, an important breathlessness, pain when swallowing, or blood in mouth, prolonged febrile neutropenia	No^b^	Feasib.	41	Cancer patients receiving CT associated with ≥ 20% overall risk of febrile neutropenia	- Feasible; high usability; high compliance; high satisfaction - Fewer unplanned hospitalizations and reduced cost of hospitalization for neutropenia compared with a historical cohort	–	Denis [35]
BREATH (Breast Cancer E-Health system)	Yes Web-based Distress reporting with cognitive behavioral therapy and include information,, assignment (48 tasks, or homework), assessment (10 self-tests followed by automated feedback), and video	Yes Web-based (No therapist involved) Self-management intervention to support the psychological adjustment	RCT (Usual psychological care + BREATH vs Usual psychological care)	150	Breast cancer survivors who had completed surgery + adjuvant CT and/or RT	- Significantly less distress and clinically significant improvement in the BREATH arm	–	Van den Berg [36]
Cankado	Yes Web-based app Symptoms self-reporting and alert function to the patient	No^b^	RCT	822 participants (CHAPLIN)	Metastatic non-squamous NSCLC or extensive-stage SCLC	–	–	NCT03911219
Care Expert	Yes Web-based. Three supportive functions: continuous communication, reinforcement of self-driven agency, and cooperative agency with a sense of being looked after	Yes Web-based	Feasib.	4	Breast receiving outpatient CT	- High usability and usefulness; high patient satisfaction related to the system’s reliability and real-time reporting function	–	Ventura [37]
CHOICE	Yes Web-based Global symptom distress reporting and provides information that is personalized and interactively tailored to patients’ specific needs, and that patients can share knowledge and experience to better manage their own care (assessment component, self-management information, communication, diary).	Yes Web-based Individually tailored information and self-management support, e-communication with expert cancer nurses	Feasib.	52	Various	- Easy to use - Availability of PROs before clinical visits led to higher congruence in addressing the symptoms during consultation	–	Ruland [38]
			Feasib.	65 nurses; 12 physicians	N/A	–	- High usefulness by nurses and physicians; higher use among nurses	Ruland [39]
			RCT (availability of PRO data vs No PRO data before consultation)	145	Patients starting antileukemia or -lymphoma treatment	- PRO data availability before visits led to 1) addressing more symptoms during consultation; 2) significant decrease of symptom distress; 3) significant reduction in need for symptom management support	–	Ruland [40]
COMPASS (Capturing and Analyzing Sensor and Self-Report Data for Clinicians and Researchers)	Yes Smartphone-based app and wearable heart rate monitor device-agnostic eHealth technology platform that can passively and remotely monitor multiple domains of function and PROs Passive monitoring of patients’ health status	No Only customizable reports to clinicians	Feasib.	3 patients; 10 HCPs	Cancer patients and HCPs	- Feasible; reassuring; highest interest in symptom monitoring	- Feasible; highest interest in monitoring of vital signs and medication adherence	Lucas [41]
*eCO* (eCediranib/Olaparib)	Yes Smartphone-based app. Blood pressure monitor linked to the app via Bluetooth and diarrhea symptom management	Yes^b^ Smartphone app and phone-based	Pilot	16	Patients with recurrent ovarian cancer enrolled in a phase II study of cediranib/olaparib (NCT 02345265)	- Feasible. High usability; high compliance; feeling of improved team-based supportive care, allowed rapid provider response and positive overall patient experience - Hypertension and diarrhea events reported at a similar frequency via *eCO* and by HCPs in the study database.	–	Liu [42]
						–	–	
eDiary	Yes Smartphone-based app Electronic daily symptom diary: severity ratings of pain, nausea, vomiting, fatigue, and sleep, other selected physical sequelae and selected descriptors of their mood	No	Feasib.	10	Adolescents and young adults with various types of cancer receiving CT	- Feasible; high usefulness; high compliance; few technical issues; very easy to use	–	Baggott [44]
						–	–	
ESRA-C (Electronic Self-report Assessment-Cancer)	Yes Web-based Self-report symptom and quality of life	Yes Web-based Self-care education and customized coaching on how to report concerns to clinicians	RCT (self-monitoring + self-care education vs self-monitoring + self-care education + coaching for communication with clinicians	752	Various. Patients starting CT or RT	- Reduced symptom distress in the intervention arm; higher benefit in > 50-year-old patients. Significantly more patients in the intervention arm reported symptoms and HRQOL during clinic visits	- No difference in clinicians’ responses between arms	Berry [45], Berry [46]
				374	Various. Patients starting CT or RT who used the tool voluntarily	- Higher use by patients starting RT - Reduced symptom distress in the intervention arm		Berry [47]
The Health Buddy® System	Yes Phone-based tele-messaging Daily response to symptom management algorithms using a simple telehealth messaging device	Yes^b^ Phone-based telemessaging Support provided to patients	Feasib.	39; 44	Newly diagnosed H&N	- Feasible, well-accepted, reassuring - System limitation: land-based phone line required	- Well accepted	Head [48, 49]
			RCT	80		- Significant improvement in QOL and lower symptom burden posttreatment. No significant improvement in social and emotional well-being - Well accepted, few technical issues		Pfeifer [50]
Home-based telehealth service	Yes PC/phone/tablet-based videoconferencing	Yes PC/phone/tablet-based video- conferencing	Feasib. (Home-based telehealth rehabilitation vs Clinic-based rehabilitation)	30	H&N after CT or RT	- Reduced number and duration of appointments until discharge - Easy to use; good audio/visual quality; high satisfaction	- Easy to use; good audio/visual quality; high satisfaction; allowed for adequate clinical assessment	Collins [51]
HRQOL in routine oncology practice	Yes Touchscreen computers Symptoms, depression scale and HRQOL questionnaire	No	RCT (Patient-reported HRQOL vs No reporting)	286 patients;28 oncologists	Various	- Improved HRQOL in intervention arm. No difference in patient-management efficiency - Improved patient-HCP communication	- Mostly rated by oncologists as “very useful” or “quite useful”	Velikova [52]
Interactive voice response (IVR) system	Yes Phone-based Patients rated symptoms twice weekly for 4 weeks via automated telephone calls. (11-point scale), 5 targeted symptoms met or exceeded a preset severity threshold. Symptoms and severity thresholds were chosen in consultation with the thoracic surgery staff.	No^b^ (email-based alert to HCPs in study arm) Email alert was forwarded to the patient’s clinical team for response if any of a subset of symptoms	RCT (IVR monitoring + clinical alerts vs IVR monitoring)	79	Primary lung or lung metastases scheduled for thoracic surgery	- Significantly fewer severe symptoms and significantly less symptom interference in the IVR + clinical alerts group - Easy to use IVR system, better rates in the IVR + clinical alerts group	- Technologically easy to implement	Cleeland [53]
IVR system	Yes Phone-based Called twice weekly by the IVR system and asked to rate the intensity of their pain and other symptoms	No (only education content) Email alerts to HCP	Pilot	60	Breast- and cancer-related pain	- Significantly greater decrease in moderate to severe pain; improvement in sleep disturbance and drowsiness	- Rated as only somewhat useful by physicians	Anderson [56]
Interaktor	Yes Web-based app Daily symptom assessment (HRQOL model) 1) regular assessment of self-reported symptoms, 2) connection to a monitoring web-interface, 3) risk assessment models for alerts, 4) continuous access to evidence-based self-care advice	Yes^b^ Self-care advice Two levels of alerts to the HCP	Randomized; in progress	150	Prostate (NCT02477137)	–	–	Langius-Eklöf [54]
				150	Breast (NCT02479607)	–	–	–
	Yes Smartphone-based app Daily symptom assessment (HRQOL model)	Yes^b^ Web-based Self-care advice Two levels of alerts to the HCP	Feasib.	6	Patients with pancreatic cancer after pancreatico-duodenectomy	- Reassuring; high compliance; easy to use	–	Gustavell [55]
						–	–	
KAIKU®	Yes Web-based app Self-assess patient side effects QOL and free text collecting PROs on early adverse effects of radiotherapy and on health-related quality of life	No^a^	Pilot	5	H&N	- Improved patient-HCP communication - Improved follow-up of patients	–	Peltola [57]
MeQoL	Yes Smartphone-based app Daily recording of degree of perceived distress, pain intensity, weekly QoL assessment, short-form 8; Minimal Documentation System.	No	Feasib.	40	Patients with solid cancer with at least monthly appointments in outpatient clinic	- Feasible; high usability; beneficial; would use again; high compliance	- Feasible; high usability; would use again -	Benze [58]
MOOVCARE™	Yes Web-based app Weekly self-scored patient symptoms (weight, weight variation, appetite loss, weakness, pain, cough, breathlessness, depression, fever, face swelling, lump under skin, voice changing, blood in sputum)	No^a^ Web-mediated follow up, weekly report and self-reported symptoms automatically triggered an alert sent to the oncologist by e-mail when predefined criteria were fulfilled.	Pilot	42	Patients with surgical excision, complete response, or non-progressive lung carcinoma	- Feasible; reassuring; reduced anxiety; high compliance - Relapses detected 5 weeks earlier with Moovcare than usual planned visits	–	Denis [59]
			Moovcare (prospective) vs Routine surveillance (retrospective)	98		- Significantly improved OS with Moovcare. High compliance	–	Denis [60]
			RCT	121	Non-progressive advanced lung	- Significantly improved OS and better performance status at relapse with Moovcare	–	Denis [61]
			Pooled analysis of 4 prospective studies (including Denis [69, 71]) vs Routine surveillance	300	Lung/various	- Significantly improved OS with Moovcare	–	Denis [62]
NCI PRO-CTCAE (STAR)	Yes Web-based (Weekly email prompt of symptom monitoring, 12 symptoms: appetite loss, constipation, cough, diarrhea, dyspnea, dysuria, fatigue, hot flashes, nausea, pain, neuropathy, and vomiting.)	No^b^ STAR triggered e-mail alerts to nurses whenever a patient-reported symptom worsened by ≥ 2 points or reached an absolute grade ≥ 3	RCT	766	Advanced solid tumors. Patients receiving outpatient CT	- Significantly improved HRQOL; fewer ER visits; fewer hospitalizations; longer time on CT. Greater clinical improvements among patients without prior computer experience	–	Basch [8]
						- Significantly increased OS	–	Basch [7]
NCI PRO-CTCAE	Yes Web- or AVR system-based (ePRO, 30 PRO-CTCAE) Self-report symptoms and physical functioning using the PRO-Core system weekly	No^b^	Feasib.	500 in PROSPECT (NCT 01515787)	Locally advanced rectal cancer	- High compliance; few technical difficulties (e.g., patient log-in issues and slow internet connectivity)	–	Basch [63]
NOONA	Yes Web-based software; can be integrated to wearable devices (www.noona.com) AE questionnaire: symptoms and distress prompted once per month and one week prior to any medically indicated oncology clinic visit.	Yes Web-based Recommendation to contact care team if required	Feasib. study in progress	100	Gastrointestinal	–	–	NCT03459352
			RCT final visit of adjuvant RT follow up by phone or Noona	765	Early breast cancer	- 40% of the patients preferred phone - 30% Noona while 30% considered both modalities equally good. - For patient choosing Noona easiness to contact. No difference in quality of life, symptoms or patient satisfaction between the modalities. Compliance was 98%	–	https://ascopubs.org/doi/abs/10.1200/JCO.2018.36.15_suppl.e18883
OASIS (Oncology Associated Symptoms and Individualized Strategies)	Yes Web-based app (https://oasis.nursing.uiowa.edu/AboutOasis) Monitoring platform to track symptom distress with educational information about cancer symptoms	Yes Web-based app Provide self-management strategies for symptoms	Feasib. In progress	56 patients; 57 caregivers; 9 HCPs	Adult potential system users from rural areas	- Easy to use; relevant content (patients and caregivers)	- Feasible; easy to use; relevant content	Gilbertson-White [64]
Oncokompas	Yes Web-based PROMs completion targeting QOL domains	Yes Web-based Tailored advice and personalized referral to supportive care options based on PROM scores and expressed preferences.	Feasib.	11	HCPs specialized in H&N cancer	–	*-Positive*: Favorable attitude of HCPs toward the eHealth application *-Negative*: Complex structure	Duman-Lubberding [65]
			Feasib.	56	H&N cancer survivors	- Feasible; high adoption and usage rates; good satisfaction with positive NPS	–	Duman-Lubberding [66]
			Feasib.	68	Breast cancer survivors who had completed surgery ± CT and/or RT	- High adoption and usage rates; good satisfaction but negative NPS - Improved patient activation but no difference in patient-HCP communication	–	Melissant [67]
			RCT in progress (Oncokompas vs Wait-list control)	544	Breast, colorectal, H&N cancer, or lymphoma survivors	–	–	Van der Hout [68]
OWise	Yes Web-based app Physical and psychological symptom registration. Information regarding type of breast cancer. Diary and calendar. Question to ask to doctor.	Yes Web-based app Personalized information and support	Feasib.		Breast	- Symptom reporting was the least-used feature; improved patient-HCP communication - Increased well-being of patients		www.owise.uk
Oxford Telemedicine System	Yes Mobile app Patients were asked to enter twice a day their temperature and symptoms: nausea, vomiting, mucositis, diarrhea/bowel movements and hand–foot syndrome(CTCAE-based)	Yes^b,c^ Mobile-based Self-care advice on their phone, directly related to their symptom. Nurses respond to alerts	Feasib.	6	Colon receiving adjuvant CT	- Reassuring; fast HCP response to alerts; patient empowerment. Overall correct generation of clinical alerts, with few false alerts generated due to missing data and poor connectivity to network	Capable and confident with the system; no work overload due to alerts	Weaver [69]
			Pilot	6	Colon receiving capecitabine	- Feasible with amber alerts generated correctly; reassuring, feeling of less “bothersome” to HCPs; high compliance	Capable and confident with the system; no work overload due to alerts	Larsen [70]
Pharmacist-run tele-oncology service for CINV monitoring	Yes Phone-based SMS system Patients’ CINV symptoms were monitored through short message service	Yes^b^ Phone-based SMS system SMS advice and call from pharmacists for uncontrolled situation	Feasib.	60	Cancer patients receiving single-day moderate to highly emetogenic chemotherapy^d^	*Positive*: Feasible; rated highly useful; high compliance *- Negative*: Dissatisfaction of patients who did not experience CINV; debatable usefulness	–	Yap [71]
Phone- or Web-based system	Yes Phone- or Web-based Depression and pain follow-up	Yes^b^ Phone- or Web-based Centralized telecare management by a nurse-physician specialist team coupled with automated home-based symptom monitoring by interactive voice recording or internet	RCT	405	Various (solid and hematologic). Patients with cancer-related pain and depression	- Significant improvement in depression and pain severity; improved HRQOL, anxiety; fewer hospital days and ER visits; no difference in disability days, physical symptoms and healthcare/co-intervention use	–	Kroenke [72, 73]
Remote monitoring and treatment (RMT) application	Yes Phone-based Severity of and change in self-reported symptoms, well-being, and daily physical activity And wearable sensor-based with: (1) a symptom and physical activity monitoring (S&PAM) system, and (2) a web-accessible exercise program (WEP) with remote supervision by a physiotherapist	No Information accessible both for patients and HCPs via a Web portal	Feasib.	22	Primary lung cancer patients scheduled for curative lung resection	- Feasible; good usability, usefulness, and satisfaction	*Positive*: Favorable perception of the exercise program *- Negative*: Low HCP perception of the added value of the symptom monitoring system	Timmerman [74]
SIS.NET (System for Individualized Survivorship Care)	Yes Web-based survey Scheduled cancer related visits to clinic. Online health questionnaires + evaluation of self-reported symptoms Short Form Health Survey (SF-36) and the 8-item Personal Health Questionnaire Depression Scale (PHQ-8), medical conditions, family history, Memorial Symptom Assessment Scale	No Notification to nurse practitioner, symptoms followed by phone as necessary	RCT	100	Breast cancer survivors	- More “new” or “changed” symptoms reported in the SIS.NET arm. No significant differences between arms in healthcare resource utilization	Nurses addressed 74% of reported new or changed patients’ symptoms within 3 days. Reasons for delayed response: 1) system malfunction; 2) problems contacting patients by phone	Wheelock [75]
SyMon-L IVR system	Yes Phone-based Patients completed questionnaires and symptom surveys via interactive voice response weekly: fatigue, poor appetite, difficulty breathing, and treatment side effects, pain, cough, shortness of breath	No^b^ (Email-based alert to HCPs in study arm) Patients’ clinically significant symptom scores generated an email alert to the site nurse for management	RCT (IVR monitoring + clinical alerts vs IVR monitoring)	153	Advanced lung	- No difference between groups in reducing symptom burden or in HRQOL - Feasible; high patient satisfaction and compliance in both groups		Yount [76]
SymptomCare@Home (SCH)	Yes Phone-based (land line) Patient has to call the automated telephone symptom-monitoring system daily: fatigue, pain, trouble in sleeping, nausea, vomiting…	Yes^a^ Web-based decision support-symptom management system; phone-based (land line) immediate automated algorithms-based self-care -management tailored to the reported symptom prevalence and severity, coaching and HCP follow-up	RCT	358	Cancer patients receiving CT	- Monitoring and reporting of 11 symptoms - Significantly lower symptom severity, fewer days of moderate and severe symptoms		Mooney [77]
			RCT (Subanalysis of Mooney [77])	252	Cancer patients with CT-induced peripheral neuropathy	- Fewer days of moderate and severe CT-induced peripheral neuropathy and symptom distress in the SCH arm		Kolb [78]
			RCT in progress	750	Cancer patients receiving CT	–		NCT 02779725
Telehealth self-management program for pain and fatigue	Yes Phone-based (telephone, text messaging) Reporting of distress related to pain and fatigue	Yes Paper-based with phone follow-up Self-management strategies	Feasib.	40	Cancer patients with previous patient-reported pain and/or fatigue	- Not feasible; low patient adoption	–	Rocque [79]
Telemonitoring system (Philips Healthcare)	Phone-based hematology analyzer device coupled to a telecommunication hub Patients were asked to analyze their own blood (capillary) and to enter temperature and symptoms and severity (based on CTCAE) for fatigue, nausea, vomiting, diarrhea, sore throat, rash and pain	No^b^ Care team alert in case of severe symptom or abnormal blood results. Message to patient to call care team.	Pilot (Self-monitoring of symptoms and vital signs vs Hospital laboratory standard)	10	Thoracic malignancy	*Positive*: Easy to use; acceptable to patients; high compliance rate; overall correct generation of clinical alerts *- Negative*: Difficulty of device use: measurements not performed as planned. However, good clinical correlation between the system and laboratory standard	–	Nimako [80]
TRSC (Therapy-Related Symptom Checklist for Adults) and TRSC-C (for children)	Yes Web-based with interactive voice response telephone Data collection through questionnaires. Conversational data collection, short response phrases indicating understanding of the reported symptom, use of open-ended questions, directed questions, review of symptoms at designated stages	No Alerts patients when the computer has informed clinicians about patient-reported symptoms.	Feasib.	282 adults; 385 children	Various	- High satisfaction Strong correlation of TRSC and TRSC-C with medical outcomes; higher HRQOL and functional status	- High satisfaction; no increase in costs	Williams [81]
Web-based app for management of postoperative symptoms	Yes Web-based app with EHR integration Real-time symptom monitoring	No^b^ Discharge instructions and queried symptoms	Feasib. RCT (App vs App + reminders [email or SMS])	35	Patients with gynecologic cancer scheduled for open surgery	- Feasible; high recruitment and completion rates; higher use in the app + reminder arm - For HRQOL, higher mental health scores and lower physical health scores in the app + reminder arm	–	Graetz [82]
WebChoice	Yes Web-based application (www.communicaretools.org). Patients could monitor their symptoms, problems, and priorities for support in physical, functional, and psychosocial dimensions	Yes Web-based Appropriate individually tailored information and self-management activities + access to other reliable Web sources, e-forum for group discussion with other patients, e-communication with expert cancer nurses	RCT (WebChoice vs Information sheet with public cancer-related websites)	325	Breast and prostate	- Symptom distress significantly lower in WebChoice arm. Better self-efficacy, HRQOL, depression, and social support with WebChoice	–	Ruland [83]
			Post hoc analysis of RCT (Ruland [45])	325	Breast and prostate	- Use of WebChoice in 63.6% of patients. Higher usage associated with a high level of computer experience and lack of comorbidities	–	Børøsund [84]
			RCT (IPPC vs WebChoice vs usual care)	167	Breast	- WebChoice vs usual care: Reduced symptom distress, anxiety, and depression; - IPPC vs usual care: Reduced depression with IPPC	- Answering patients’ e-messages perceived as not too time consuming	Børøsund [85]
Web portal for physical activity and symptom tracking	Yes Web-based and linked to a wearable activity monitor device Collection of PROs and symptom information, symptom and health related QoL tracking	Yes Web-based Provision of educational material, and individualized coaching messaging. Remote monitoring of physical activity for patient and clinician	Feasib.	49	Various	- Feasible. Highest compliance when access to Web portal was accompanied by weekly activity reports and personalized coaching messaging	–	Marthick [86]

^a^For RCTs, the digital health tool was compared with usual care, unless otherwise specified

^b^System alerts to HCPs generated if clinically relevant symptoms were reported

^c^Red alerts for severe side effects; amber alerts for less-severe symptoms

^d^Defined by the National Comprehensive Cancer Network antiemesis guidelines v.1.2011.AE, adverse event

*ASyMS* Advanced Symptom Management System, *CINV* chemotherapy-induced nausea and vomiting, *CT* chemotherapy, *CTCAE* Common Terminology Criteria for Adverse Events, *EHR* electronic health record, *ER* emergency room, *Feasib.* feasibility, *H&N* head and neck, *HCP* healthcare professional, *HRQOL* health-related quality of life, *IPPC* internet-based patient-provider communication, *misc.* miscellaneous, *N/A* not applicable, *NCI* National Cancer Institute, *NPS* net promoter score, *OS* overall survival, *PC* personal computer, *PROs* patient-reported outcomes, *PROMs* patient-reported outcome measures, *RCT* randomized controlled trial, *RT* radiotherapy, *SMS* short message service

### Clinical evidence for adoption of digital solutions

Clinical evidence for digital health solutions evaluated in feasibility or randomized controlled studies are also summarized in Table 1 [7, 8, 25–86].

#### Drivers and barriers to usage

From the patient perspective, some of the key factors identified for the uptake of the digital tools included the following:

1. Ease of use [30, 38, 44, 51, 53, 55, 64, 80];
2. Reassurance [28, 30, 41, 48, 49, 55, 59, 70];
3. High usability and usefulness [37, 42, 44, 58, 62, 74];
4. Improved communication with HCPs [27, 29, 30, 53, 58]www.owise.uk;
5. Correct generation of system alerts and fast response to alerts [28, 70, 80];
6. Patient empowerment [29, 30, 69]; and
7. The convenience of real-time reporting of symptoms [28, 37];

One study evaluating the extent of patient use of a Web-based intervention reported that reduction of cancer symptom distress was a key driver of uptake, with use of the intervention resulting in a significant reduction in distress score [47].

Conversely, some of the barriers for adoption encountered by patients were as follows:

1. Problems with technology or connectivity [48, 49, 69, 80];
2. Limited usefulness [29, 30, 71];
3. Lack of clarity of the language used [29, 30]; and
4. Generation of false alerts [69].

Whereas higher education level, current employment, and low levels of social support have been associated with uptake, lower education level and non-working status may be barriers to accessing interventions [47, 84].

Fewer studies have assessed the feasibility of digital solutions from the HCP perspective. The most important reasons for adoption reported by HCPs were the usability and usefulness of the tool [26, 38, 52, 58], and the most commonly reported barrier was problems with technology or connectivity [31, 75].

Interestingly, while some tools were perceived as a burden due to increased workload [28], others did not impact the working time of HCPs [69, 85].

#### Impact on clinical assessment

Most studies presented ePRO data, including symptom distress and burden, pain, depression, and adherence.

A meta-analysis of 9 studies reported a statistically significant benefit for digital interventions in patients with cancer-related fatigue, with moderate benefits also observed for QOL and depression [45].

Several studies showed a significant reduction compared with usual care in symptom-related distress on the basis of measures that included Short-Form (SF)-36, Memorial Symptom Assessment Scale (MSAS), Symptom Distress Scale-15 (SDS-15), and Functional Assessment of Cancer Therapy-Head & Neck Scale (FACT-HN) [36, 40, 45, 47, 50, 78, 85]. Symptom benefit was observed in conjunction with automated home or Web-based symptom self-management systems.

Studies also reported a reduction in depression [73, 85], symptom severity [33, 53], pain [43, 56, 73, 77], and need for symptom management support [40].

An RCT enrolling 766 patients with solid tumors receiving outpatient chemotherapy demonstrated that self-reporting of 12 common cancer-related symptoms led to significant improvement in QOL, as measured by the EuroQol EQ-5D Index [8].

Two studies used the European Organization for Research and Treatment of Cancer Qualify of Life Questionnaire Core 30 (EORTC-QLQ-C30) for QOL assessment [43, 52]. One of these used the EORTC-QLQ-C30 and the Hospital Anxiety and Depression Scale (HADS) as an intervention, with a larger proportion of patients who reported these measures to their oncologists showing clinically meaningful improvements in QOL compared with a control group, despite no detectable changes in patient management [52].

An RCT evaluating the impact of an internet-based exercise intervention reported significant improvement in EORTC-QLQ-C30 scores for global health status, physical, role, and cognitive functioning, together with improvements in pain severity on the Brief Pain Inventory compared with control [43].

In another study of a Web-based intervention, the addition of self-care instructions and communication coaching to Electronic Self-report Assessment–Cancer (ESRA-C) of symptoms and QOL resulted in significant increase in reporting fatigue, pain, and physical function issues. However, differences between groups in symptom distress reported by patient did not reach significance [46].

Finally, a report found benefit for patient QOL, including increased symptom identification and management, and improved functional status following electronic collection of Therapy-Related Symptom Checklist for Adults (TRSC) [81].

#### Impact on survival

A prospective study compared survival in patients with lung cancer who were assigned to weekly symptom self-reporting via a Web application intervention for early detection of relapse with a retrospective group of control patients [60]. Median OS was improved for the patients assigned to the intervention compared with the historical control arm.

Survival outcomes were also reported in 2 RCTs. A single-center trial reported that integration of ePROs into the routine care of patients with metastatic cancer led to increased survival compared with usual care [7]. At a median follow-up of 7 years, median OS was 31.2 months (95% CI, 24.5–39.6) in the group that provided self-report of 12 common symptoms, with severe or worsening symptoms triggering an email alert and follow-up care by a nurse practitioner with escalation as needed. In comparison, median OS in the group assigned to usual care was 26.0 months (95% CI, 22.1–30.9; difference, 5 months; *P* = .03). In patients with advanced lung cancer, a multicenter study reported that intervention involving a Web-based follow-up algorithm to assess weekly patient symptom self-reports compared with routine follow-up resulted in median OS of 19.0 (95% CI, 12.5-noncalculable) and 12.0 months (95% CI, 8.6–16.4), respectively (*P =* .001) [61]. In addition, the performance status at first relapse was 0 to 1 for 76% of patients in the intervention arm compared with 33% in the control arm (2-sided *P* < .001); anticancer treatment was considered to be optimal in 72% and 33%, respectively (2-sided *P* < .001). In the final OS analysis for this study, median OS was 22.5 months in the intervention group and 14.9 months in the control group (hazard ratio, 0.59 [95% CI, 0.37–0.96]; *P* = .03) [87].

#### Impact on ER admissions, hospitalizations, and healthcare resource utilization

The effect of digital solutions on the number of ER visits, hospital days, or utilization of healthcare resources is not commonly evaluated in clinical studies. Some solutions, involved in patient monitoring providing or not providing feedback for self-management, have been associated with a reduction in ER visits, unplanned hospitalizations, and hospital days [8, 35, 73]. Additionally, use of a telehealth system for rehabilitation of patients with head and neck cancer following chemo-/radiotherapy resulted in fewer and shorter appointments until discharge compared with usual care and was accompanied by a significant cost-reduction for patients, specifically in travel costs [51]. On the contrary, one study using a Web-based intervention that included review by a nurse practitioner found no differences compared with control with respect to healthcare resource use, including oncology-related appointments, number of physician visits, or medical tests [75]. The effect of digital solutions on overall healthcare costs needs further assessment [8, 35, 73, 75].

### Clinical benefits and limitations of the digital solutions for stakeholders

**Fig. 3:**
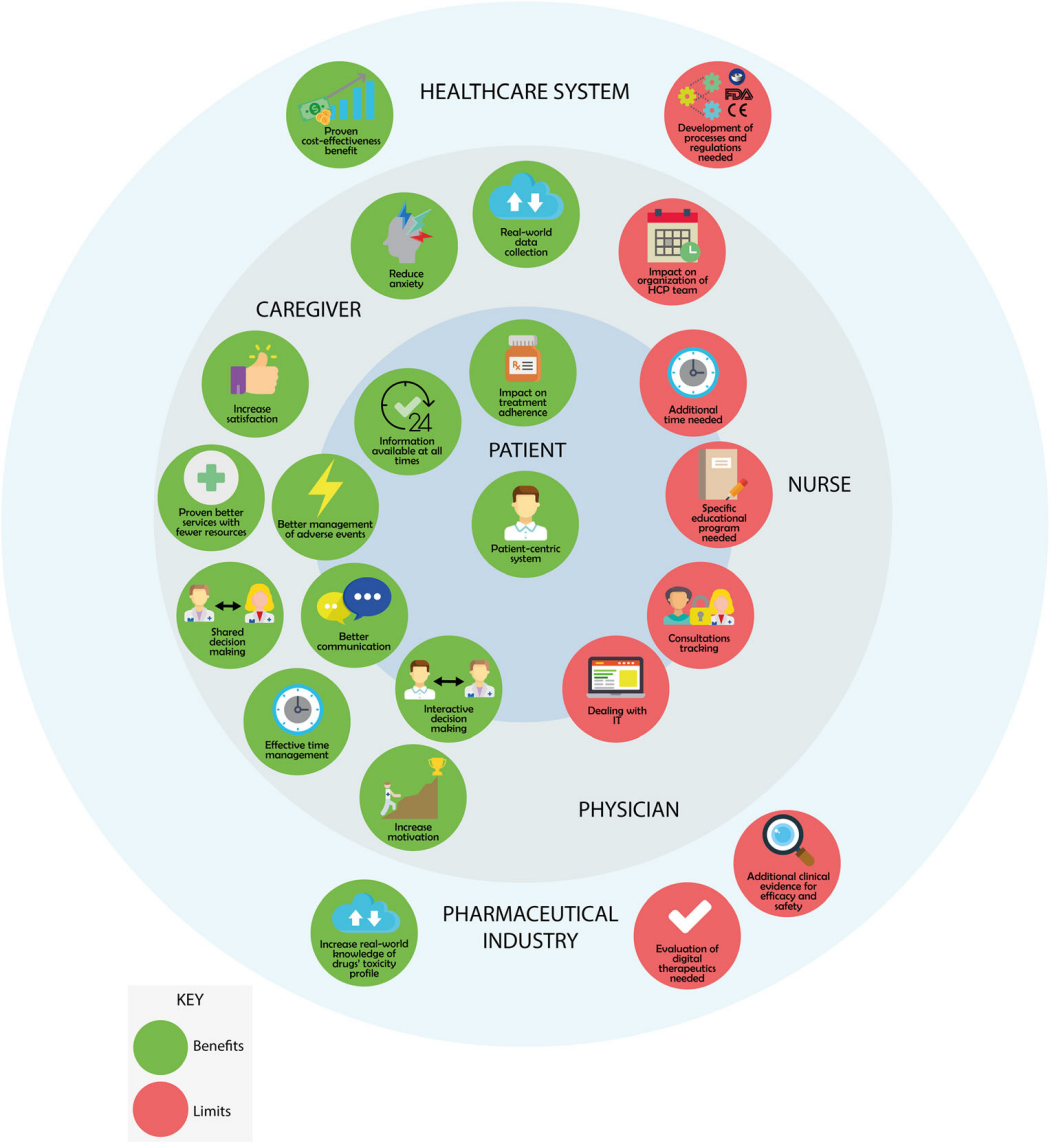
Benefits and limitations of digital solutions in the healthcare system. FDA US Food and Drug Administration, HCP healthcare professional, IT information technology

Benefits and limitations of introducing a patient-management solution in oncology, according to stakeholders of digital solutions in the healthcare system, are summarized in Table 2 and illustrated in Fig. 3. These benefits and limitations were identified in the selected publications and from the authors experience and opinion. Lots of benefits have been identified of important impact on all stakeholders (patients, physicians, caregivers, nurses, healthcare system, pharmaceutical company), with limitations related to technical dealing, regulatory constraints, costs, and changes in practices.

**Table 2:** Benefits and limitations of digital healthcare solutions for stakeholders

Stakeholder	Benefits	Limitations
Patients	- Promote patient-centricity - Direct communication with HCPs - Closer involvement in the decision-making process - Impact on treatment-adherence - Information from clinical visits always available - Relevant disease- and treatment-related information always available - Less recourse to generic Web consultation without scientific content	- Difficulty in dealing with technology - Need for specific education and training - Time-consuming - Uncomfortable asking clinicians for permission to record clinical visits - Depersonalization
Physicians	- Improved communication with patients - Shared decision-making by involving patients in the process - Real-world data collection in real time - Optimal management of toxicities in real time • Increased motivation thanks to visible improvements - Effective time-management • Time saving in the analyses of patients’ data • Contact patients only when clinically relevant situations occur - Focused supportive care - Less healthcare resource utilization	- Difficulty in dealing with technology - Need for specific training to ensure engagement - Time dedicated outside of consultation hours - Changes in the organization of HCP teams - Difficulty in changing usual practices of symptom management
Nurses	- Effective time-management • Time saving in the analyses of patients’ data • Contact patients only when clinically relevant situations occur - Increased quality of services with less healthcare resource utilization - Improved patient-nurse communication	- Difficulty in dealing with technology - Need for specific training to ensure engagement - Time dedicated to educating and inform patients and caregivers - Additional time allocated outside patients’ visits
Caregivers	- Reduced burden and anxiety - Increased satisfaction	- Difficulty in dealing with technology - Need for specific education and training
Healthcare system	- Impact of preventive care in healthcare costs. Cost-effectiveness benefits • Reduction in ER visits, wait time in ER, transportation costs • Reduction in unplanned visits and hospitalizations • Impact on the working time of physicians, nurses, ER personnel • Reduction in medication cost • Prevention and treatment of AEs more consistent with guidelines	- Need for development of processes and regulations for homologation of digital solutions by regulatory agencies - Formation and training of dedicated teams for evaluation - Delays in cost-effectiveness analyses for the implementation of reimbursement policies, resulting in impeded access to patients
Pharmaceutical industry	- Real-world data and increased knowledge of the toxicity profile of drugs - Development of plans for improved management of AEs - Expedited approval of drugs when filing in combination with digital solutions	- Additional studies with the drug + digital solution combination needs to be performed, to generate clinical evidence of efficacy and safety to support filing: increased time and cost

*AE* adverse event, *ER* emergency room, *HCP* healthcare professional

## Discussion

Although the clinical benefits of remote patient monitoring have been demonstrated in clinical trials [7, 62], achieving optimal supportive care requires strategies that go beyond ePRO apps/systems. Such benefits are not obtained solely through the assessment of outcomes of interest but also through appropriate management in response to assessments. Even if benefits have been confirmed in the setting of RCTs, there is a need to continue to evaluate ePRO efficacy and efficiency in real-world conditions, with ongoing assurances of data security and privacy, to provide relevant information for optimal self-management.

Several factors need to be considered for a high-quality symptom self-management system. Guidance from the treating physician is critical. Electronic self-reported assessment tools for cancer-related symptoms and QOL can increase communication between patients and HCPs and promote discussion that is focused on symptoms and QOL. Digital tools that give advice to patients on the reporting of symptoms to HCPs have been shown to increase symptom reports by patients during visits. However, these have not been shown to impact practitioner responses, indicating that guideline adherence and commitment by the medical team is also needed. The collection of information regarding related clinical symptoms and the medication received requires integration with electronic real-time monitoring of symptoms into oncologists’ routine clinical practice. When real-time monitoring is used, beneficial outcomes in terms of symptom management have been identified [88], with the potential for further optimization when structured patient education or practitioner-/nurse-led symptom counseling is in place. Optimization of digital tools requires integration with the patients’ EHRs, thereby allowing continuity in the flow of patient-related data and the healthcare support systems.

Digital health solutions need to be integrated into the patient pathway and in healthcare team practices for optimal supportive care in oncology in line with appropriate guidelines. How this integration is implemented is debatable, with consideration given as to whether the digital tool is merged into current healthcare systems in a gradual or disruptive manner. The European Society for Medical Oncology (ESMO) has developed a Magnitude of Clinical Benefit Scale (ESMO-MCBS) to assess the extent of the clinical benefit from new and effective anticancer therapies measuring improvement in survival, disease-free survival, response, grade 3–4 toxicities, and QOL measures [89]. MCBS-based assessment of the digital tools as part of anticancer therapies and the use of MCBS for the development of clinical guidelines would ease this integration.

There are challenges in the development of a digital solution for supportive care of cancer patients. Setting up and conducting clinical trials for the evaluation of digital tools is a long process, especially because digital solutions need to be quickly available for evaluation in real-world settings. The principal difficulties are in developing and implementing a solution to fit the needs of all or most patients, while achieving the necessary patient compliance to change with the new digital tool and integrate it into care and maintaining enough adaptability for its use in different regulatory systems and healthcare centers. Implementation may be associated with challenges in staff having to deal with new technologies, accepting and adapting to changes, and the potential for reorganization of multidisciplinary teams/treatment centers. Maintenance of the device may also introduce complexity since device utility is dependent on updates in accordance with relevant guidelines, as well as drug safety information, approval of new drugs, and the use of different drugs from the same class. Oncologic therapy is by its nature complex, with sequential phases, and device utility will need to reflect the use of different antitumor regimens, including radiotherapy and radio-chemotherapy, and combination of drugs. Uptake of the technology may be dependent on oncologist perceptions of patients’ willingness to adopt new technologies, as well as the actual willingness of patient subgroups, particularly elderly patients, to embrace digital solutions. Finally, digital solutions should be perceived as facilitators of in-person communication between patient and practitioner.

This review offers elements for scoping digital solution based on feasibility studies on limited level of evidence or still limited numbers of patients evaluated on RCT.

## Outlook for the future

**Fig. 4:**
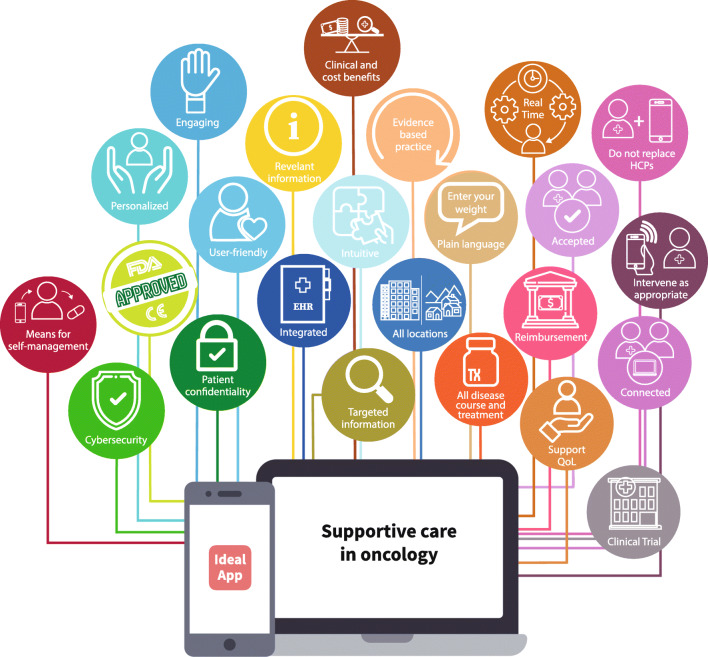
Ideal digital health solution

Several clinical studies have already demonstrated reliability, feasibility, and clinical value (various symptoms, QOL, and OS) with efficacy of ePRO collection through digital solutions. The ideal digital solution in the setting of supportive care in oncology would present with the following characteristics (Fig. 4): it would be user-friendly, intuitive, and engaging to meet the immediate needs of the end-users; it would also be efficient at processing and delivering relevant information to provide supportive care as its principal aim. In thinking about its place in the supportive care setting, the ideal digital solution is not intended as a replacement for the practitioner; rather, its intended value would be in providing additional information that is appropriate to the care of the patient and the specific issues associated with their disease in real time. This information would be sufficiently detailed but not overcomplicated and presented in a language the patient understands in order to be accessible by the patient for effective symptom self-management [90]. The digital solution would maintain existing expectations regarding patient confidentiality and data privacy [91], cybersecurity, compliance with regulatory requirements, and being updated according to the most recent evidence-based practice. It would be operational throughout the entire course of the disease and for all anticancer treatments. Its built-in flexibility would enable adaptation of the digital tool to all territories, institutions, and centers and to all different care needs according to whether treatment is delivered in the community or at a regional center, such that it also serves patients who live in remote areas. It would be customizable to adapt to the needs of the individual patient. It would have a seamless connection with HCPs’ systems. Integration with patients’ EHR would allow for rapid follow-up and intervention as appropriate by HCPs in response to system alerts triggered by patient reports of clinically relevant events. It would have a high level of acceptance both by HCPs and patients, allowing its complete adoption and full integration in the patient pathway and in routine clinical practice. For digital solutions with proven clinical and cost benefits, reimbursement policies would be in place to ensure availability for implementation through defined market access programs. Finally, the ideal digital solution would not only provide the means for patient self-management of anticancer treatment-related symptoms but would also provide psychosocial support and improve QOL. Although a single system would not be able to address all needs—treatment adherence, symptom management, alignment with guidelines, medication reminders, medical and nutritional information, resources for social support, and coping strategies—it is important that digital tools find common ground, with solutions offered to address key challenges in the setting of supportive care in cancer.

## References

[1] Ferlay J, Colombet M, Soerjomataram I, Mathers C, Parkin DM, Piñeros M, Znaor A, Bray F (2019). Estimating the global cancer incidence and mortality in 2018: GLOBOCAN sources and methods. Int J Cancer.

[2] Allemani C, Matsuda T, Di Carlo V (2018). Global surveillance of trends in cancer survival 2000-14 (CONCORD-3): analysis of individual records for 37 513 025 patients diagnosed with one of 18 cancers from 322 population-based registries in 71 countries. Lancet.

[3] Ferlay J, Soerjomataram I, Dikshit R, Eser S, Mathers C, Rebelo M, Parkin DM, Forman D, Bray F (2015). Cancer incidence and mortality worldwide: sources, methods and major patterns in GLOBOCAN 2012. Int J Cancer.

[4] Cook R (2008). Economic and clinical impact of multiple myeloma to managed care. J Manag Care Pharm.

[5] Jordan K, Aapro M, Kaasa S, Ripamonti CI, Scotté F, Strasser F, Young A, Bruera E, Herrstedt J, Keefe D, Laird B, Walsh D, Douillard JY, Cervantes A (2018). European Society for Medical Oncology (ESMO) position paper on supportive and palliative care. Ann Oncol.

[6] Kaasa S, Loge JH, Aapro M, Albreht T, Anderson R, Bruera E, Brunelli C, Caraceni A, Cervantes A, Currow DC, Deliens L, Fallon M, Gómez-Batiste X, Grotmol KS, Hannon B, Haugen DF, Higginson IJ, Hjermstad MJ, Hui D, Jordan K, Kurita GP, Larkin PJ, Miccinesi G, Nauck F, Pribakovic R, Rodin G, Sjøgren P, Stone P, Zimmermann C, Lundeby T (2018). Integration of oncology and palliative care: a Lancet Oncology Commission. Lancet Oncol.

[7] Basch E, Deal AM, Dueck AC, Scher HI, Kris MG, Hudis C, Schrag D (2017). Overall survival results of a trial assessing patient-reported outcomes for symptom monitoring during routine cancer treatment. JAMA.

[8] Basch E, Deal AM, Kris MG, Scher HI, Hudis CA, Sabbatini P, Rogak L, Bennett AV, Dueck AC, Atkinson TM, Chou JF, Dulko D, Sit L, Barz A, Novotny P, Fruscione M, Sloan JA, Schrag D (2016). Symptom monitoring with patient-reported outcomes during routine cancer treatment: a randomized controlled trial. J Clin Oncol.

[9] Beaton L, Bandula S, Gaze MN, Sharma RA (2019). How rapid advances in imaging are defining the future of precision radiation oncology. Br J Cancer.

[10] Kaufman HL, Atkins MB, Subedi P, Wu J, Chambers J, Joseph Mattingly T, Campbell JD, Allen J, Ferris AE, Schilsky RL, Danielson D, Lichtenfeld JL, House L, Selig WKD (2019). The promise of immuno-oncology: implications for defining the value of cancer treatment. J Immunother Cancer.

[11] Galligioni E, Piras EM, Galvagni M, Eccher C, Caramatti S, Zanolli D, Santi J, Berloffa F, Dianti M, Maines F, Sannicolò M, Sandri M, Bragantini L, Ferro A, Forti S (2015). Integrating mHealth in oncology: experience in the province of Trento. J Med Internet Res.

[12] Shah CP, Moreb JS (2019). Cardiotoxicity due to targeted anticancer agents: a growing challenge. Ther Adv Cardiovasc Dis.

[13] McCarthy PL, Holstein SA, Petrucci MT (2017). Lenalidomide maintenance after autologous stem-cell transplantation in newly diagnosed multiple myeloma: a meta-analysis. J Clin Oncol.

[14] Partridge AH, Wang PS, Winer EP, Avorn J (2003). Nonadherence to adjuvant tamoxifen therapy in women with primary breast cancer. J Clin Oncol.

[15] Barquín-García A, Molina-Cerrillo J, Garrido P, Garcia-Palos D, Carrato A, Alonso-Gordoa T (2019). New oncologic emergencies: what is there to know about immunotherapy and its potential side effects?. Eur J Intern Med.

[16] American Society of Clinical Oncology. The state of cancer care in America, 2016: a report by the American Society of Clinical Oncology. 2016. 10.1200/jop.2015.010462. Accessed September 24, 2019 10.1200/JOP.2015.010462 PMC5015451 26979926

[17] Meskó B, Drobni Z, Bényei É, Gergely B, Győrffy Z (2017). Digital health is a cultural transformation of traditional healthcare. Mhealth.

[18] Kruse CS, Goswamy R, Raval Y, Marawi S (2016). Challenges and opportunities of big data in health care: a systematic review. JMIR Med Inform.

[19] Schneeweiss S, Eichler HG, Garcia-Altes A, Chinn C, Eggimann AV, Garner S, Goettsch W, Lim R, Löbker W, Martin D, Müller T, Park BJ, Platt R, Priddy S, Ruhl M, Spooner A, Vannieuwenhuyse B, Willke RJ (2016). Real-world data in adaptive biomedical innovation: a framework for generating evidence fit for decision-making. Clin Pharmacol Ther.

[20] van Roessel I, Reumann M, Brand A (2017). Potentials and challenges of the health data cooperative model. Public Health Genomics.

[21] Raposo VL. Telemedicine: the legal framework (or the lack of it) in Europe. GMS Health Technol Assess 2016; 12: Doc03 10.3205/hta000126 PMC4987488 27579146

[22] Rajkomar A, Dean J, Kohane I (2019). Machine learning in medicine. N Engl J Med.

[23] Fallowfield LJ (2018). Quality of life assessment using patient-reported outcome (PRO) measures: still a Cinderella outcome?. Ann Oncol.

[24] Aapro M, Molassiotis A, Dicato M, Peláez I, Rodríguez-Lescure Á, Pastorelli D, Ma L, Burke T, Gu A, Gascon P, Roila F, PEER investigators (2012). The effect of guideline-consistent antiemetic therapy on chemotherapy-induced nausea and vomiting (CINV): the Pan European Emesis registry (PEER). Ann Oncol.

[25] Kearney N, McCann L, Norrie J (2009). Evaluation of a mobile phone-based, advanced symptom management system (ASyMS) in the management of chemotherapy-related toxicity. Support Care Cancer.

[26] Maguire R, McCann L, Miller M, Kearney N (2008). Nurse’s perceptions and experiences of using a mobile-phone-based Advanced Symptom Management System (ASyMS) to monitor and manage chemotherapy-related toxicity. Eur J Oncol Nurs.

[27] McCann L, Maguire R, Miller M, Kearney N (2009). Patients’ perceptions and experiences of using a mobile phone-based advanced symptom management system (ASyMS) to monitor and manage chemotherapy related toxicity. Eur J Cancer Care (Engl).

[28] Maguire R, Ream E, Richardson A, Connaghan J, Johnston B, Kotronoulas G, Pedersen V, McPhelim J, Pattison N, Smith A, Webster L, Taylor A, Kearney N (2015). Development of a novel remote patient monitoring system: the advanced symptom management system for radiotherapy to improve the symptom experience of patients with lung cancer receiving radiotherapy. Cancer Nurs.

[29] Breen S, Kofoed S, Ritchie D, Dryden T, Maguire R, Kearney N, Aranda S (2017). Remote real-time monitoring for chemotherapy side-effects in patients with blood cancers. Collegian.

[30] Breen S, Ritchie D, Schofield P (2015). The Patient Remote Intervention and Symptom Management System (PRISMS) – a Telehealth-mediated intervention enabling real-time monitoring of chemotherapy side-effects in patients with haematological malignancies: study protocol for a randomised controlled trial. Trials.

[31] Furlong E, Darley A, Fox P, Buick A, Kotronoulas G, Miller M, Flowerday A, Miaskowski C, Patiraki E, Katsaragakis S, Ream E, Armes J, Gaiger A, Berg G, McCrone P, Donnan P, McCann L, Maguire R (2019). Adaptation and implementation of a mobile phone-based remote symptom monitoring system for people with cancer in Europe. JMIR Cancer.

[32] Maguire R, Fox PA, McCann L, Miaskowski C, Kotronoulas G, Miller M, Furlong E, Ream E, Armes J, Patiraki E, Gaiger A, Berg GV, Flowerday A, Donnan P, McCrone P, Apostolidis K, Harris J, Katsaragakis S, Buick AR, Kearney N (2017). The eSMART study protocol: a randomised controlled trial to evaluate electronic symptom management using the advanced symptom management system (ASyMS) remote technology for patients with cancer. BMJ Open.

[33] Spoelstra SL, Given BA, Given CW, Grant M, Sikorskii A, You M, Decker V (2013). An intervention to improve adherence and management of symptoms for patients prescribed oral chemotherapy agents: an exploratory study. Cancer Nurs.

[34] Low CA, Dey AK, Ferreira D, Kamarck T, Sun W, Bae S, Doryab A (2017). Estimation of symptom severity during chemotherapy from passively sensed data: exploratory study. J Med Internet Res.

[35] Denis F, Voog E, Pointreau Y, Bourgeois H, Seegers V, le du K (2019). Prospective study of a web-mediated management of febrile neutropenia related to chemotherapy (Bioconnect). Support Care Cancer.

[36] van den Berg SW, Gielissen MF, Custers JA (2015). BREATH: web-based self-management for psychological adjustment after primary breast cancer--results of a multicenter randomized controlled trial. J Clin Oncol.

[37] Ventura F, Koinberg I, Sawatzky R, Karlsson P, Öhlén J (2016). Exploring the person-centeredness of an innovative e-supportive system aimed at person-centered care: prototype evaluation of the care expert. Comput Inform Nurs.

[38] Ruland CM, White T, Stevens M, Fanciullo G, Khilani SM (2003). Effects of a computerized system to support shared decision making in symptom management of cancer patients: preliminary results. J Am Med Inform Assoc.

[39] Ruland CM (2006). Clinicians’ perceived usefulness of a support system for patient-centered cancer care. Stud Health Technol Inform.

[40] Ruland CM, Holte HH, Røislien J (2010). Effects of a computer-supported interactive tailored patient assessment tool on patient care, symptom distress, and patients’ need for symptom management support: a randomized clinical trial. J Am Med Inform Assoc.

[41] Lucas AR, Bass MB, Rothrock NE, O'Connor ML, Sorkin MR, Nawyn J, Albinali F, Wagner LI (2018). Development of an eHealth system to capture and analyze patient sensor and self-report data: mixed-methods assessment of potential applications to improve cancer care delivery. JMIR Med Inform.

[42] Liu JF, Lee JM, Strock E, Phillips R, Mari K, Killiam B, Bonam M, Milenkova T, Kohn EC, Ivy SP (2018). Technology applications: use of digital health technology to enable drug development. JCO Clin Cancer Inform.

[43] Galiano-Castillo N, Cantarero-Villanueva I, Fernández-Lao C, Ariza-García A, Díaz-Rodríguez L, del-Moral-Ávila R, Arroyo-Morales M (2016). Telehealth system: a randomized controlled trial evaluating the impact of an internet-based exercise intervention on quality of life, pain, muscle strength, and fatigue in breast cancer survivors. Cancer.

[44] Baggott C, Gibson F, Coll B, Kletter R, Zeltzer P, Miaskowski C (2012). Initial evaluation of an electronic symptom diary for adolescents with cancer. JMIR Res Protoc.

[45] Berry DL, Hong F, Halpenny B, Partridge AH, Fann JR, Wolpin S, Lober WB, Bush NE, Parvathaneni U, Back AL, Amtmann D, Ford R (2014). Electronic self-report assessment for cancer and self-care support: results of a multicenter randomized trial. J Clin Oncol.

[46] Berry DL, Hong F, Halpenny B, Partridge A, Fox E, Fann JR, Wolpin S, Lober WB, Bush N, Parvathaneni U, Amtmann D, Ford R (2014). The electronic self-report assessment and intervention for cancer: promoting patient verbal reporting of symptom and quality of life issues in a randomized controlled trial. BMC Cancer.

[47] Berry DL, Blonquist TM, Patel RA (2015). Exposure to a patient-centered, Web-based intervention for managing cancer symptom and quality of life issues: impact on symptom distress. J Med Internet Res.

[48] Head BA, Studts JL, Bumpous JM, Gregg JL, Wilson L, Keeney C, Scharfenberger JA, Pfeifer MP (2009). Development of a telehealth intervention for head and neck cancer patients. Telemed J E Health.

[49] Head BA, Keeney C, Studts JL, Khayat M, Bumpous J, Pfeifer M (2011). Feasibility and acceptance of a telehealth intervention to promote symptom management during treatment for head and neck cancer. J Support Oncol.

[50] Pfeifer MP, Keeney C, Bumpous J, Schapmire T, Studts J, Myers J, Head B (2015). Impact of a telehealth intervention on quality of life and symptom distress in patients with head and neck cancer. J Community Support Oncol.

[51] Collins A, Burns CL, Ward EC, Comans T, Blake C, Kenny L, Greenup P, Best D (2017). Home-based telehealth service for swallowing and nutritional management following head and neck cancer treatment. J Telemed Telecare.

[52] Velikova G, Booth L, Smith AB, Brown PM, Lynch P, Brown JM, Selby PJ (2004). Measuring quality of life in routine oncology practice improves communication and patient well-being: a randomized controlled trial. J Clin Oncol.

[53] Cleeland CS, Wang XS, Shi Q, Mendoza TR, Wright SL, Berry MD, Malveaux D, Shah PK, Gning I, Hofstetter WL, Putnam JB, Vaporciyan AA (2011). Automated symptom alerts reduce postoperative symptom severity after cancer surgery: a randomized controlled clinical trial. J Clin Oncol.

[54] Langius-Eklöf A, Crafoord MT, Christiansen M, Fjell M, Sundberg K (2017). Effects of an interactive mHealth innovation for early detection of patient-reported symptom distress with focus on participatory care: protocol for a study based on prospective, randomised, controlled trials in patients with prostate and breast cancer. BMC Cancer.

[55] Gustavell T, Langius-Eklöf A, Wengström Y, Segersvärd R, Sundberg K (2019). Development and feasibility of an interactive smartphone app for early assessment and management of symptoms following pancreaticoduodenectomy. Cancer Nurs.

[56] Anderson KO, Palos GR, Mendoza TR, Cleeland CS, Liao KP, Fisch MJ, Garcia-Gonzalez A, Rieber AG, Nazario LA, Valero V, Hahn KM, Person CL, Payne R (2015). Automated pain intervention for underserved minority women with breast cancer. Cancer.

[57] Peltola MK, Lehikoinen JS, Sippola LT (2016). A novel digital patient-reported outcome platform for head and neck oncology patients-a pilot study. Clin Med Insights Ear Nose Throat.

[58] Benze G, Nauck F, Alt-Epping B, Gianni G, Bauknecht T, Ettl J, Munte A, Kretzschmar L, Gaertner J (2019). PROutine: a feasibility study assessing surveillance of electronic patient reported outcomes and adherence via smartphone app in advanced cancer. Ann Palliat Med.

[59] Denis F, Viger L, Charron A, Voog E, Dupuis O, Pointreau Y, Letellier C (2014). Detection of lung cancer relapse using self-reported symptoms transmitted via an internet web-application: pilot study of the sentinel follow-up. Support Care Cancer.

[60] Denis F, Yossi S, Septans AL, Charron A, Voog E, Dupuis O, Ganem G, Pointreau Y, Letellier C (2017). Improving survival in patients treated for a lung cancer using self-evaluated symptoms reported through a web application. Am J Clin Oncol.

[61] Denis F, Lethrosne C, Pourel N, Molinier O, Pointreau Y, Domont J, Bourgeois H, Senellart H, Trémolières P, Lizée T, Bennouna J, Urban T, el Khouri C, Charron A, Septans AL, Balavoine M, Landry S, Solal-Céligny P, Letellier C (2017) Randomized trial comparing a web-mediated follow-up with routine surveillance in lung cancer patients. J Natl Cancer Inst 109 10.1093/jnci/djx029 28423407

[62] Denis F, Basch EM, Lethrosne C (2018). Randomized trial comparing a web-mediated follow-up via patient-reported outcomes (PRO) vs. routine surveillance in lung cancer patients: final results. J Clin Oncol.

[63] Basch E, Dueck AC, Rogak LJ et al (2018) Feasibility of implementing the patient-reported outcomes version of the Common Terminology Criteria for Adverse Events in a multicenter trial: NCCTG N1048. J Clin Oncol 36. 10.1200/JCO.2018.78.8620 10.1200/JCO.2018.78.8620 PMC6209091 30204536

[64] Gilbertson-White S, Yeung CW, Saeidzadeh S, Tykol H, Vikas P, Cannon A (2019). Engaging stakeholders in the development of an eHealth intervention for cancer symptom management for rural residents. J Rural Health.

[65] Duman-Lubberding S, van Uden-Kraan CF, Peek N, Cuijpers P, Leemans CR, Verdonck-de Leeuw IM (2015). An eHealth application in head and neck cancer survivorship care: health care professionals’ perspectives. J Med Internet Res.

[66] Duman-Lubberding S, van Uden-Kraan CF, Jansen F, Witte BI, van der Velden LA, Lacko M, Cuijpers P, Leemans CR, Verdonck-de Leeuw IM (2016). Feasibility of an eHealth application “OncoKompas” to improve personalized survivorship in cancer care. Support Care Cancer.

[67] Melissant HC, Verdonck-de Leeuw IM, Lissenberg-Witte BI, Konings IR, Cuijpers P, van Uden-Kraan CF (2018). ‘Oncokompas’, a web-based self-management application to support patient activation and optimal supportive care: a feasibility study among breast cancer survivors. Acta Oncol.

[68] van der Hout A, van Uden-Kraan CF, Witte BI, Coupé VMH, Jansen F, Leemans CR, Cuijpers P, van de Poll-Franse LV, Verdonck-de Leeuw IM (2017). Efficacy, cost-utility and reach of an eHealth self-management application ‘Oncokompas’ that helps cancer survivors to obtain optimal supportive care: study protocol for a randomised controlled trial. Trials.

[69] Weaver A, Young AM, Rowntree J, Townsend N, Pearson S, Smith J, Gibson O, Cobern W, Larsen M, Tarassenko L (2007). Application of mobile phone technology for managing chemotherapy-associated side-effects. Ann Oncol.

[70] Larsen ME, Rowntree J, Young AM (2008). Chemotherapy side-effect management using mobile phones. Conf Proc IEEE Eng Med Biol Soc.

[71] Yap KY, Low HX, Koh KS (2013). Feasibility and acceptance of a pharmacist-run tele-oncology service for chemotherapy-induced nausea and vomiting in ambulatory cancer patients. Telemed J E Health.

[72] Kroenke K, Theobald D, Norton K, Sanders R, Schlundt S, McCalley S, Harvey P, Iseminger K, Morrison G, Carpenter JS, Stubbs D, Jacks R, Carney-Doebbeling C, Wu J, Tu W (2009). The Indiana Cancer Pain and Depression (INCPAD) trial design of a telecare management intervention for cancer-related symptoms and baseline characteristics of study participants. Gen Hosp Psychiatry.

[73] Kroenke K, Theobald D, Wu J, Norton K, Morrison G, Carpenter J, Tu W (2010). Effect of telecare management on pain and depression in patients with cancer: a randomized trial. JAMA.

[74] Timmerman JG, Dekker-van Weering MGH, Stuiver MM (2017). Ambulant monitoring and web-accessible home-based exercise program during outpatient follow-up for resected lung cancer survivors: actual use and feasibility in clinical practice. J Cancer Surviv.

[75] Wheelock AE, Bock MA, Martin EL, Hwang J, Ernest ML, Rugo HS, Esserman LJ, Melisko ME (2015). SIS.NET: A randomized controlled trial evaluating a web-based system for symptom management after treatment of breast cancer. Cancer.

[76] Yount SE, Rothrock N, Bass M, Beaumont JL, Pach D, Lad T, Patel J, Corona M, Weiland R, del Ciello K, Cella D (2014). A randomized trial of weekly symptom telemonitoring in advanced lung cancer. J Pain Symptom Manag.

[77] Mooney KH, Beck SL, Wong B, Dunson W, Wujcik D, Whisenant M, Donaldson G (2017). Automated home monitoring and management of patient-reported symptoms during chemotherapy: results of the symptom care at home RCT. Cancer Med.

[78] Kolb NA, Smith AG, Singleton JR, Beck SL, Howard D, Dittus K, Karafiath S, Mooney K (2018). Chemotherapy-related neuropathic symptom management: a randomized trial of an automated symptom-monitoring system paired with nurse practitioner follow-up. Support Care Cancer.

[79] Rocque GB, Halilova KI, Varley AL, Williams CP, Taylor RA, Masom DG, Wright WJ, Partridge EE, Kvale EA (2017). Feasibility of a telehealth educational program on self-management of pain and fatigue in adult cancer patients. J Pain Symptom Manag.

[80] Nimako K, Lu SK, Ayite B, Priest K, Winkley A, Gunapala R, Popat S, O'Brien MER (2013). A pilot study of a novel home telemonitoring system for oncology patients receiving chemotherapy. J Telemed Telecare.

[81] Williams AR, Williams DD, Williams PD, Alemi F, Hesham H, Donley B, Kheirbek RE (2015). The development and application of an oncology therapy-related symptom checklist for adults (TRSC) and children (TRSC-C) and e-health applications. Biomed Eng Online.

[82] Graetz I, Anderson JN, McKillop CN (2018). Use of a web-based app to improve postoperative outcomes for patients receiving gynecological oncology care: a randomized controlled feasibility trial. Gynecol Oncol.

[83] Ruland CM, Andersen T, Jeneson A, Moore S, Grimsbø GH, Børøsund E, Ellison MC (2013). Effects of an internet support system to assist cancer patients in reducing symptom distress: a randomized controlled trial. Cancer Nurs.

[84] Børøsund E, Cvancarova M, Ekstedt M, Moore SM, Ruland CM (2013). How user characteristics affect use patterns in web-based illness management support for patients with breast and prostate cancer. J Med Internet Res.

[85] Børøsund E, Cvancarova M, Moore SM, Ekstedt M, Ruland CM (2014). Comparing effects in regular practice of e-communication and web-based self-management support among breast cancer patients: preliminary results from a randomized controlled trial. J Med Internet Res.

[86] Marthick M, Dhillon HM, Alison JA, Cheema BS, Shaw T (2018). An interactive web portal for tracking oncology patient physical activity and symptoms: prospective cohort study. JMIR Cancer.

[87] Denis F, Basch E, Septans AL, Bennouna J, Urban T, Dueck AC, Letellier C (2019). Two-year survival comparing web-based symptom monitoring vs routine surveillance following treatment for lung cancer. JAMA.

[88] Strasser F, Blum D, von Moos R, Cathomas R, Ribi K, Aebi S, Betticher D, Hayoz S, Klingbiel D, Brauchli P, Haefner M, Mauri S, Kaasa S, Koeberle D (2016). The effect of real-time electronic monitoring of patient-reported symptoms and clinical syndromes in outpatient workflow of medical oncologists: E-MOSAIC, a multicenter cluster-randomized phase III study (SAKK 95/06). Ann Oncol.

[89] Cherny NI, Dafni U, Bogaerts J, Latino NJ, Pentheroudakis G, Douillard JY, Tabernero J, Zielinski C, Piccart MJ, de Vries EGE (2017). ESMO-magnitude of clinical benefit scale version 1.1. Ann Oncol.

[90] Atema V, van Leeuwen M, Oldenburg HSA, van Beurden M, Hunter MS, Aaronson NK (2017). An internet-based cognitive behavioral therapy for treatment-induced menopausal symptoms in breast cancer survivors: results of a pilot study. Menopause.

[91] Alberts NM, Hadjistavropoulos HD, Dear BF, Titov N (2017). Internet-delivered cognitive-behaviour therapy for recent cancer survivors: a feasibility trial. Psychooncology.

